# A targeted promotional DVD fails to improve Māori and Pacific participation rates in the New Zealand bowel screening pilot: results from a pseudo-randomised controlled trial

**DOI:** 10.1186/s12889-019-7582-7

**Published:** 2019-09-09

**Authors:** Karen Bartholomew, Lifeng Zhou, Sue Crengle, Elizabeth Buswell, Anne Buckley, Peter Sandiford

**Affiliations:** 1 Waitematā District Health Board, 15 Shea Terrace, Takapuna, Auckland, 0740 New Zealand; 2 Department of Preventive and Social Medicine, Dunedin School of Medicine, University of Otago, Dunedin, 9054 New Zealand; 3 School of Population Health, University of Auckland, Private Bag 92019, Auckland, 1142 New Zealand

**Keywords:** Bowel cancer screening, Small media interventions, Māori, Pacific, Faecal immunochemical test, Colorectal cancer, Screening participation, Ethnic inequalities, Equity

## Abstract

**Background:**

New Zealand’s Bowel Screening Pilot (BSP) used a mailed invitation to return a faecal immunochemical test. As a pilot it offered opportunities to test interventions for reducing ethnic inequities in colorectal cancer screening prior to nationwide programme introduction. Small media interventions (e.g. educational material and DVDs) have been used at both community and participant level to improve uptake. We tested whether a DVD originally produced to raise community awareness among the Māori population would have a positive impact on participation and reduce the proportion of incorrectly performed tests (spoiled kits) if mailed out with the usual reminder letter.

**Methods:**

The study was a parallel groups pseudo-randomised controlled trial. Over 12 months, all Māori and Pacific ethnicity non-responders four weeks after being mailed the test kit were allocated on alternate weeks to be sent, or not, the DVD intervention with the usual reminder letter. The objective was to determine changes in participation and spoiled kit rates in each ethnic group, determined three months from the date the reminder letter was sent. Participants and those recording the outcomes (receipt of a spoiled or non-spoiled test kit) were blinded to group assignment.

**Results:**

2333 Māori and 2938 Pacific people participated (11 withdrew). Those who were sent the DVD (1029 Māori and 1359 Pacific) were less likely to participate in screening than those who were not (1304 Māori and 1579 Pacific). Screening participation was reduced by 12.3% (95% CI 9.1–15.5%) in Māori (13.6% versus 25.9%) and 8.3% (95% CI 5.8–10.8%) in Pacific (10.1% versus 18.4%). However, spoiled kit rates (first return) were significantly higher among those not sent the DVD (33.1% versus 12.4% in Māori and 42.1% versus 21.9% in Pacific).

**Conclusion:**

The DVD sent with the reminder letter to BSP non-responders reduced screening participation to an extent that more than offset the lower rate of spoiled kits.

**Trial registration:**

Australia and New Zealand Clinical Trials Registry ACTRN12612001259831. Registered 30 November 2013.

**Electronic supplementary material:**

The online version of this article (10.1186/s12889-019-7582-7) contains supplementary material, which is available to authorized users.

## Background

Waitematā District Health Board (DHB) is responsible for a population of approximately 600,000 people in the north and west of Auckland, New Zealand (NZ). Waitematā DHB conducted a pilot of a Bowel Screening programme (BSP) from 2012 to 2017 to inform the potential introduction of an organised colorectal cancer screening programme in NZ [1]. The BSP mailed an immunohistochemical Faecal Occult Blood (iFOBT) self-test kit to all male and female Waitematā residents aged 55–74 years on the BSP population register, which was based on the National Health Index (a unique identifier given to all users of publicly funded health services). The pilot pathway comprised a pre-invitation letter, mailed self-test kit four weeks later, self-test completion, and return of results (via the participant’s general practitioner if positive). Non-participation resulted in a reminder letter being sent four weeks after the iFOBT was sent. For Māori, Pacific and Asian ethnicity invitees, a phone call follow-up was made concurrently with the reminder letter.

Strategies to optimise ethnic-specific participation in screening programmes are of international interest. Lessons on successful strategies from well-established screening programmes such as breast and cervical screening have been applied to colorectal cancer screening [2, 3]. A variety of interventions for colorectal cancer screening, in both organised and opportunistic settings, have been examined across patient, provider, organisation and policy intervention levels [4–6]. Interventions intended to increase participation at the patient level have emphasised the provision of ‘small media’ (health education or promotional materials e.g. brochures, booklets, video/DVD) in the context of screening invitation and follow up of non-participation.

The rationale for small media intervention is to act on social cognitive variables such as knowledge or attitude, aiming to reduce barriers such as risk perception or fear of the test [7]. While there is good evidence that small media does improve knowledge and facilitates a more favourable attitude to screening tests, there is equivocal evidence of the link between this and improved screening participation [4]. Many studies have demonstrated that some educational material has a positive impact on screening participation (variable range of effect depending on the kind of intervention and the setting) [2, 3]. There is also evidence that the provision of more detailed risk or test based information reduces screening participation (although informed consent may be increased) [8].

The BSP offered an opportunity to test whether a targeted bowel cancer screening health promotion DVD would raise participation and/or reduce the rate of incorrectly performed tests (referred to henceforth as the spoiled kit rate). The DVD focused on the indigenous Māori population and was developed locally for community awareness raising activities. This study examines its impact on Māori and Pacific screening participation when utilised as an adjunct to the usual non-participation reminder letter.

## Methods

### DVD development

A locally produced six-minute promotional DVD was developed as a tool for Māori community awareness-raising as part of a suite of activities aimed at increasing Māori BSP participation. That is, it was not developed as an adjunct to the bowel screening test kit process. The DVD was intended to be viewed in a range of community settings. A famous Māori rugby player delivered key programme messages aimed at improving knowledge and reducing barriers, including the ease and cleanliness of the test, and key features of invitation and programme participation. The DVD also featured two well-known local Māori elders presenting a narrative description of their programme participation experience. This narrative emphasised the importance of the test, the ease of the test, the nature of return of results and the positive experience of the diagnostic follow-up test. Although the intended audience were Māori, it was felt that many Pacific people would also be able to identify with the experiences portrayed by the protagonists.

### Study participants

Māori and Pacific ethnic group’s participation in the first year of the BSP was considerably lower than other ethnic groups (including Asian, NZ European and Other ethnicities) the DVD intervention was tested in both groups. In New Zealand ethnicity is self-identified and respondents may select multiple ethnicities. For both invitation and analysis prioritised ethnicity was used where, in accordance with Ministry of Health Standards, Māori overrides all other ethnicities, Pacific overrides all but Māori and Asian ethnicity is recorded in priority to European [9, 10].

For a 12-month period from 4 January 2013 to 31 December 2013, all Māori and Pacific people who were enrolled in the BSP but did not return a test kit within the usual programme interval of four weeks were classified as non-responders and included in the study.

### Intervention and control groups

The intervention consisted of including the promotional DVD with the reminder letter sent out to people who had not responded (non-responders) to the initial screening invitation. The control group was comprised of non-responders who were only sent the usual reminder letter.

### Group allocation

Following the pilot programme’s existing procedures, reminder letters were generated in batches 3–4 times a week for eligible individuals (Māori and Pacific) once the time from the date of being sent an invitation had exceeded 4 weeks without a test kit being returned to the Coordination Centre. All Māori and Pacific participants were identified for each batch of reminder letters manually. Participants with both Māori and Pacific ethnicity were assigned to the Māori group in accordance with the prioritised ethnicity procedures described above. Study participants were allocated to the DVD plus reminder letter or reminder letter only groups on alternate weeks. DVDs required a slightly larger envelope than the reminder letter alone.

Recruitment monitoring performed approximately halfway through the trial revealed an imbalance in group accrual with more individuals being assigned to the intervention than the control group. This was found to be mainly due to the timing of batches for the reminder letters. To correct the imbalance, the weeks assigned for intervention and control were reversed at week 35 in the study.

There was also some inconsistency in whether the participants were correctly sent DVDs or not according to their DVD/no DVD week allocation according to records kept of who was sent a DVD. This resulted in a slightly lower proportion being sent a DVD than should have been the case if the protocol had been followed correctly (45.3% versus 49.3%). This was mainly due to allocation of patients who became eligible during weekends. To test for any systematic bias a logistic regression model compared those who were misallocated (i.e. incorrectly sent DVDs in a ‘non-DVD week’ or not sent a DVD in a ‘DVD week’) with those who were correctly sent DVDs or not according to their allocated week. This found no significant differences between them in ethnicity, gender, age or deprivation quintile. On this basis it was concluded that the misallocation was completely at random and we conducted all analyses on a per protocol basis. The logistic regression results and a separate analysis on an ‘intention to randomise’ basis is provided as Additional file 1.

### Outcomes

There were two pre-specified primary outcomes of interest for the study: the difference in participation and the difference in spoiled kit return associated with being sent the DVD. Participation in the BSP was defined by the return of a test kit regardless of the result (positive, negative or ‘spoiled kit’). Participation was censored at three months post reminder letter generation in accordance with BSP standard operating procedure. ‘Spoiled’ kits are samples which cannot be analysed for one reason or another (e.g. no date on the specimen). A return was considered spoiled if any of the kits returned by that individual were spoiled. Those returning spoiled kits were sent replacement kits with an explanation as to why the previous sample was not usable.

Two non-prespecified secondary outcomes were analysed: the ‘Good first kit’ return rate (first kit returned was not spoiled hence the number of spoiled kits was zero) and the ‘Any good kit’ return rate (a good kit was returned at some stage and hence a test result was obtained).

### Analysis

The trial was powered to detect a 5% difference in participation between intervention and control groups in both primary outcomes. Self-identified ethnicity was sourced from the BSP Register (ethnicity as recorded on the National Health Index). Prioritised ethnicity was used in analyses. Area deprivation was determined using the New Zealand Index of Deprivation 2006 (NZDep06) and grouped into quintiles from most deprived (Quintile 5) to least deprived (Quintile 1) [11, 12]. Participants who withdrew from the BSP within 3 months of the DVD/reminder letter were excluded from the analysis.

Wilson 95% confidence limits were calculated for all proportions and differences between proportions by Newcombe’s method without continuity correction [13, 14]. Separate pre-specified analyses were performed for Māori and Pacific invitees since it was hypothesised that the DVD might have more impact on Māori than Pacific ethnicity recipients. The Fleiss test of homogeneity of risk differences was used to assess whether a pooled Māori and Pacific analysis would be valid [15]. A log-binomial regression model was fitted to assess whether the impact of the DVD on outcomes changed after controlling for potential confounding by age, sex and deprivation. A separate model was fitted for Māori and Pacific. Data was analysed in Excel®, SAS® 9.4 and Stata® 13.

## Results

**Fig. 1:**
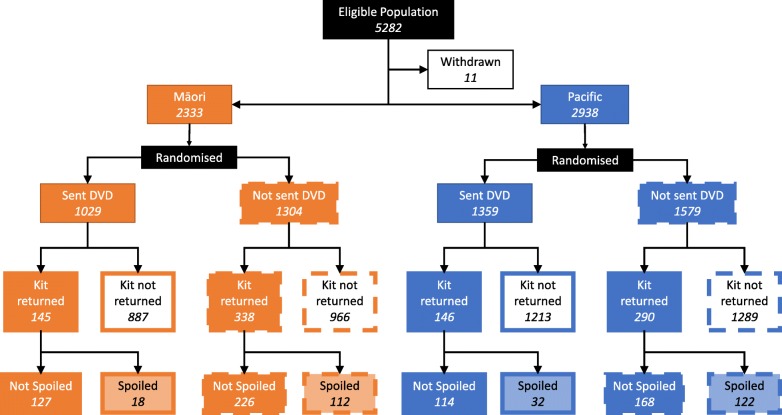
Consort Diagram

After 12 months 5271 people (2333 Māori and 2938 Pacific) were randomised (see Consort Diagram in Fig. 1). Of those 2388 were sent a DVD with the reminder letter and 2883 just the usual reminder letter, the difference being attributable to the initial imbalance in group allocation resulting from the pseudo-randomisation process as described above. Table 1 shows that the DVD group did not differ from the no DVD group in demographic characteristics that are known to influence screening participation overall (ethnicity, age, sex and deprivation).

**Table 1:** Demographic characteristics of those who were sent the DVD compared with those who were not

	Māori					Pacific				
	*N*	Sent DVD	Not sent DVD	Sent DVD %	p*	*N*	Sent DVD	Not sent DVD	Sent DVD %	p*
Gender					0.08					0.60
Male	939	390	549	41.5%		1304	589	715	45.2%	
Female	1377	622	755	45.2%		1604	740	864	46.1%	
Missing	17	17	0			30	30	0		
Age					0.24					0.92
50–54	909	413	496	45.4%		995	459	536	46.1%	
55–59	642	270	372	42.1%		732	326	406	44.5%	
60–64	391	178	213	45.5%		560	263	297	47.0%	
65–69	249	106	143	42.6%		365	164	201	44.9%	
70+	125	45	80	36.0%		256	117	139	45.7%	
Missing	17	17	0			30	30	0		
Deprivation Quintile					0.34					0.80
Q1 (least)	284	110	174	38.7%		155	71	84	45.8%	
Q2	348	158	190	45.4%		256	112	144	43.8%	
Q3	454	195	259	43.0%		515	227	288	44.1%	
Q4	603	274	329	45.4%		964	453	511	47.0%	
Q5 (most)	402	183	219	45.5%		754	349	405	46.3%	
Missing	242	109	133			294	147	147		
Total	2333	1029	1304	44.1%		2938	1359	1579	46.3%	

*Chi square probability testing for no difference in the proportions sent DVD. Missing values were excluded from the significance tests

Table 2 presents results from bivariate kit return (participation) analysis. It shows that for Māori, bowel cancer screening participation was 25.9% in the No DVD group and 13.6% in the DVD group. This is a 12.3% absolute reduction in participation and a 48.4% relative reduction in screening participation for Māori who were sent the DVD compared with Māori who did not. For the Pacific ethnicity group participation was 18.4% for the No DVD group and 10.1% for the DVD group, a 8.3% absolute and 45.1% relative reduction in participation. The Fleiss test for homogeneity of the risk difference rejected the null hypothesis (*p* = 0.043) which demonstrates that there was a significantly stronger absolute reduction in participation associated with being sent a DVD in Māori (compared with Pacific) that was more than should be expected by chance. For this reason, pooled analysis of kit return rates for two ethnic groups is not presented in Table 2. In a non-prespecified analysis it heterogeneity was detected between Māori males and females in kit return rates (*p* = 0.035); this was of borderline significance for Pacific (*p* = 0.075), with a stronger negative effect on participation observed in males than females. The table shows that being sent a DVD had a larger negative effect on participation among males than females.

**Table 2:** Kit return in DVD and No DVD groups by ethnicity and sex

	DVD Group	Kit returned	Kit not returned	Total	Kit return rate	Difference (95% C.I.)
Māori	Sent DVD	138	874	1012	13.6%	12.3% (9.1–15.5%)
	Not sent DVD	338	966	1304	25.9%	
Pacific	Sent DVD	134	1195	1329	10.1%	8.3% (5.8–10.8%)
	Not sent DVD	290	1289	1579	18.4%	
Māori male	Sent DVD	52	338	390	13.3%	16.4% (11.1–21.3%)
	Not sent DVD	163	386	549	29.7%	
Māori female	Sent DVD	86	536	622	13.8%	9.4% (5.2–13.4%)
	Not sent DVD	175	580	755	23.2%	
Pacific male	Sent DVD	55	534	589	9.3%	10.8% (7.0–14.5%)
	Not sent DVD	144	571	715	20.1%	
Pacific female	Sent DVD	79	661	740	10.7%	6.2% (2.8–9.6%)
	Not sent DVD	146	718	864	16.9%	

Note: 47 individuals with missing sex data are excluded from this analysis. Pooled Māori and Pacific results are not shown for the kit return outcome as the Fleiss test for homogeneity of rate difference excluded the null hypothesis

Analysis of the spoiled kit rate is presented in Table 3. The spoiled kit rate for Māori who returned a test kit was 12.4% in the DVD group compared with 33.1% in those who were not sent a DVD, an absolute difference of 20.7% and a relative reduction in kit spoilage associated with being sent a DVD of 62.5%. The corresponding figures for the Pacific group were 21.9% kit spoilage in the DVD group, and 42.1% in the No DVD group with a risk difference of 20.2% and a relative reduction associated with the DVD of 47.9%. In this case the Fleiss test for homogeneity of risk difference was not significant (*p* = 0.92), suggesting that although Pacific had higher spoiled kit rates, the effect of the DVD on (reducing) the spoiled kit rate was similar for Māori and Pacific, and hence a pooled analysis is presented in Table 2 for this outcome, where the combined risk difference was 20.1%.

**Table 3:** Comparison of spoiled kit rates between the DVD and the usual participant reminder letter groups by ethnicity

	DVD group	Spoiled kit rate outcome				
		Spoiled kit	Not spoiled kit	Total	Spoiled kit rate	Difference (95% C.I.)
Māori	Sent DVD	18	127	145	12.4%	20.7% (13.4–28.1%)
	Not sent DVD	112	226	338	33.1%	
Pacific	Sent DVD	32	114	146	21.9%	20.2% (11.4–28.9%)
	Not sent DVD	122	168	290	42.1%	
Both	Sent DVD	50	241	291	17.2%	20.1% (14.3–25.8%)
	Not sent DVD	234	394	628	37.3%	

Given that return rates were higher without the DVD, but spoiled kit rates were lower with the DVD, a secondary analysis to test whether there was an offset in benefit was conducted. This secondary analysis compared the proportions in each group where a non-spoiled kit was received on the first return (Additional file 1: Table S2). In Māori these were 11.9% among those sent the DVD and 17.3% among those who were not sent it (difference 5.5%; 95% CI 2.6–8.3%) and in Pacific they were 7.9 and 10.6% respectively (difference 2.7%; 95% CI 0.1–4.8%). These differences are considerably smaller than those based on all kit returns, but remain significant, showing that the DVD was associated with a lower rate of non-spoiled kit return. If subsequent non-spoiled kits are included in the calculation, then the differences widen again as some of those who initially returned a spoiled kit subsequently returned a non-spoiled one. In this case the (any) non-spoiled kit return rates in Māori were 13.4% among those sent the DVD and 22.2% in those not sent it (difference 8.8, 95% CI 5.6–11.8%) and for Pacific 9.6% in the DVD group and 14.5% in the No DVD group (difference 4.9%, CI 2.5–7.2%).

The log-binomial regression analysis (Table 4) established that the significant association between being sent a DVD and lower bowel screening participation was not confounded by gender, age or deprivation for either Māori or Pacific ethnic groups. The reduction in participation associated with the DVD was very similar to that found in the bivariate analysis (46.0% vs 45.6 and 44.9% vs 41.5% respectively for Māori and Pacific). The lower Spoiled kit rate among those sent the DVD was also unaffected by controlling for gender, age and deprivation. In the log-binomial regression model the DVD was associated with 60.0% lower spoiled kit rate for Māori compared with 62.5% in bivariate analysis; for Pacific the corresponding figures were 49.7 and 47.9%.

**Table 4:** Log-binomial regression models of outcomes

	Variable	Kit return rate outcome				Spoiled kit rate outcome		
		Relative risk	Confidence limits	P		Relative risk	Confidence interval	*p*
Māori	Sent DVD	0.54	0.45–0.65	< 0.001		0.40	0.25–0.63	< 0.001
	Sex (male)	1.22	1.05–1.43	0.011		1.10	0.83–1.45	0.514
	Age Group			0.351				0.072
	50–54	1.00				1.00		
	55–59	1.03	0.85–1.25	0.771		0.89	0.64–1.22	0.457
	60–64	1.17	0.94–1.46	0.149		0.66	0.42–1.05	0.077
	65–69	0.95	0.72–1.26	0.730		0.50	0.26–0.97	0.040
	70+	1.27	0.93–1.74	0.136		0.48	0.21–1.10	0.083
	Deprivation quintile			< 0.001				0.589
	Q1 (least)	1.00				1.00		
	Q2	0.82	0.65–1.04	0.110		0.98	0.66–2.17	0.950
	Q3	0.66	0.52–0.84	0.001		1.39	0.58–1.66	0.161
	Q4	0.48	0.37–0.61	< 0.001		1.24	0.88–2.19	0.381
	Q5 (most)	0.46	0.35–0.62	< 0.001		1.38	0.77–2.11	0.209
	Missing	0.58	0.43–0.78	< 0.001		1.20	0.83–2.30	0.543
	Constant	0.35	0.29–0.43	< 0.001		0.32	0.21–0.49	< 0.001
Pacific	Sent DVD	0.55	0.46–0.67	< 0.001		0.50	0.35–0.71	< 0.001
	Sex (male)	1.09	0.91–1.30	0.318		1.02	0.79–1.32	0.863
	Age Group			0.072				
	50–54	1.00				1.00		
	55–59	1.05	0.84–1.43	0.674		1.04	0.73–1.47	0.844
	60–64	1.29	0.99–1.72	0.044		1.05	0.73–1.52	0.785
	65–69	1.40	1.01–1.86	0.014		1.05	0.71–1.55	0.808
	70+	1.21	0.88–1.67	0.242		1.20	0.79–1.83	0.384
	Deprivation quintile			< 0.001				
	Q1 (least)	1.00				1.00		
	Q2	0.53	0.36–0.78	0.001		0.72	0.35–1.48	0.373
	Q3	0.50	0.36–0.69	< 0.001		1.08	0.62–1.87	0.788
	Q4	0.49	0.37–0.66	< 0.001		1.26	0.77–2.07	0.364
	Q5 (most)	0.53	0.40–0.72	< 0.001		1.31	0.79–2.17	0.296
	Missing	0.35	0.23–0.53	< 0.001		0.92	0.44–1.91	0.817
	Constant	0.30	0.22–0.40	< 0.001		0.35	0.21–0.57	< 0.001

Note: Age group was a significant predictor of test kit return as a continuous variable for Pacific but not Māori and a significant predictor of non-Spoiled kit return in Māori but not Pacific (results not shown)

## Discussion

Small media interventions are often developed and delivered in a health setting assuming that they will have a positive impact (or at least do no harm) without any rigorous evaluation of their impact. They may be developed for population-wide application or tailored to a target audience. Tailored approaches relevant to improving ethnic-specific screening participation using small media include: use of culturally informed narratives; promotional messages delivered by group members; messages targeting particular health beliefs; use of culturally appropriate language and visual appeal; or targeted health literacy approaches [16–18]. There is some evidence that favours visual or video-based strategies both as a community awareness raising strategy (increased community demand) and a patient focused strategy [19–22]. The evidence base for tailored approaches suggests they work best as part of a multi-level approach in organised screening programmes, including phone follow-up or navigator-based approaches [23–25].

In the Waitematā DHB bowel cancer screening pilot, which used a mailed test kit, people chose to participate at home with little or no interaction with their healthcare provider. An invitee’s decision is likely to have been influenced by the written information accompanying the test.

The BSP Steering Group fully expected that the DVD would have a positive impact on screening participation in Māori and possibly Pacific despite being designed for community awareness raising. At worst it was thought the DVD might have no impact on participation. This view was consistent with the evidence base for the impact on participation of small media which supports a positive effect [4, 26], with studies showing either no difference or a positive effect of up to 14% specifically with video/DVD material [19, 20, 27]. The finding that the DVD led to lower participation was therefore disappointing and quite unexpected for the BSP Steering Group (although the result is not inconsistent with previously published research on small media interventions which has highlighted the risks of ‘information overload’). It was therefore a salient lesson regarding the importance of rigorous evaluation, even of apparently innocuous interventions, to avoid these becoming an established component of healthcare delivery in the absence of prior evidence for their efficacy.

In retrospect several possible causes for the reduced participation in the DVD group can be identified: potential participants may have found the DVD itself off-putting (with or without viewing it); some may have viewed the DVD and found the content problematic; and others possibly viewed the DVD and made an informed decision not to participate on the basis of their increased knowledge. There is a well-known relationship between the provision of more information and reduced participation in screening [28–31]. An example of this was the 16% reduction in participation seen in an Australian trial of a colorectal screening decision-aid intervention for socioeconomically disadvantaged populations [8]. However, most reports in the literature of reduced screening participation from small media interventions relate to lengthy educational booklets or intensive decision-aid techniques aimed at improving knowledge and informed consent which were not the focus of this DVD.

Though the content of the DVD may not have been ideal for use as a reminder adjunct, it is difficult to see how it would have led to the observed negative effect on screening participation. It incorporated both narrative and educational components to improve knowledge and reduce barriers to participation. The protagonist in the narrative was a woman, which may have discouraged some men, but the observed reduction in participation was significant for both sexes. This approach was advocated for, and endorsed by Māori health providers at the outset of the BSP [32].

Rather than being due to the content of the DVD, a more plausible reason for lower participation in those sent the DVD could be that some of these individuals felt that it was necessary to watch the DVD before performing the test. If they did not own a DVD player or did not have the time to watch it this would have represented a barrier to participation. No reason for providing the DVD was included with the reminder letter.

The lower rate of spoiled kits in those sent the DVD is difficult to interpret. Whereas it is tempting to attribute this to guidance provided within the DVD on how to perform the test, it is also possible that with a higher kit return rate, the additional participants in the *No DVD* group were individuals who had (unmeasured) characteristics that made them more likely to return a spoiled kit. Among those who returned kits, the *No DVD* group had a higher proportion of males than the *DVD* group (49% vs 39%), but the DVD attributable reduction in rate of spoiled kits was not significantly different between males and females (19.7% vs 19.8%). Other demographic factors did not vary greatly between the groups either. The spoiled kit rate in the BSP was very high initially (and at the time that this study was conducted). Further reductions in the spoiled kit rate were seen after a redesign of the test kit instructions and accompanying materials that was undertaken after this project finished.

### Limitations

The pseudo-randomised trial design was a convenient, efficient and practical approach in the context of an ongoing real-world programme, but in retrospect randomisation at the level of the individual might have avoided some of the problems noted in the methods section such as the unequal accrual rate in the *DVD* and *No DVD* group (although we have shown that it is unlikely to have introduced any bias – see Additional file 1 for details).

In this study we were unable to examine the reasons for reduced participation or record which *DVD* recipients watched the video. The literature suggests that other DVD media have relatively low uptake in terms of audience viewing with reasons provided by patients including lack of time, fear of cancer and not having a DVD player [33]. DVD viewing was found to be higher and related to improved screening participation in physician-based opportunistic colorectal cancer screening settings which is unlikely to be relevant to the BSP [21].

## Conclusion

It was anticipated that the targeted nature of the DVD in this study would have no effect or could produce a small marginal gain in screening participation for Māori, and potentially Pacific people in the context of the BSP. It was also hypothesised that it might lead to a reduction in spoiled kits, which at the outset of the BSP was a significant problem. Instead we found that the DVD intervention was associated with lower screening participation in both these priority groups, whose participation was already below the rest of the eligible population. Although the DVD was also associated with a reduced spoiled kit rate, this was not sufficient to offset the lower participation so that even the return rate for non-spoiled kits was lower in the intervention group. This study adds to the literature on the impact of specific types of small media intervention and it provides a reminder that the potential for harm in interventions can easily be overlooked if these are not subject to rigorous assessment prior to introduction as part of routine practice.

## Additional file

Additional file 1: Supplementary material. (DOCX 41 kb)

## Data Availability

The datasets used and/or analysed during the current study are available from the corresponding author on reasonable request.

## Abbreviations

BSP: Bowel Screening Pilot
DHB: District Health Board
DVD: Digital Video Disc
