# Supplementary material for: A targeted promotional DVD fails to improve Māori and Pacific participation rates in the New Zealand bowel screening pilot: results from a pseudo-randomised controlled trial
Source: BMC Public Health. 2019 Sep 9;19:1245. doi: 10.1186/s12889-019-7582-7 (PMC6734461; doi:10.1186/s12889-019-7582-7)
Supplement: Supplementary file 1 — Supplementary material. (DOCX 41 kb) [file 12889_2019_7582_MOESM1_ESM.docx]

Additional file 1

# Explanation for the Differential Accrual Rate

Differential accrual was found to be mostly explained by a previously undetected alternate week letter generation differential. This was attributed to the base BSP invitation approach which used date-of-birth to invite potential participants. When the background invitation number were analysed it was found that the weeks where four invitation batches were produced was more common on alternate weeks due to batches falling systematically on the weekend and being posted early in the week. Because this study was based on the non-response reminder letters batched in the same way as the initial invitation, this alternate week differential magnified the group assignment differential in favour of not receiving a DVD. Other factors which may also have accounted for the differential were also investigated. The counting of people who were ‘on hold’ in the BSP in the usual response letter group and not the DVD group was also considered a potential explanation for some of the group accrual differential. ‘On hold’ means that potential participants had contacted the BSP and indicated their intention to participate but that they would like to delay participation, for example due to an overseas holiday or moving to a new house. Participation for the two groups was recorded by the BSP using two different data sources; the *DVD group* were recorded by the team responsible for posting the letters (actual DVDs included), whereas the *No DVD* group were accounted for by the BSP invitation database which included ‘on hold’ participants. Because of these findings the week assignment was reversed at week 35. Although this resulted in a convergence of group accrual there was still a residual group differential which may have been eliminated entirely if the reversal had taken place exactly half way through the project.

# Logistic regression model predicting misallocation of intervention

**Table S1** presents odd ratios and their confidence limits for the logistic regression model of intervention misallocation where a DVD was either not sent in a week allocated for DVD or sent in a week allocated to ‘No DVD’. Subjects with missing age (n=47) were excluded from the analysis. There was a greater proportion of not sent DVDs in ‘DVD weeks’ (12.9%) than DVDs sent in ‘No DVD’ weeks (4.6%). No other variables significantly predicted misallocation although deprivation was of borderline significance.

**Table S1**. Logistic regression of potential predictors of intervention misallocation

| **Variable** | **Odds ratio** | **(95% C.I.)** | **Z ratio** | **Prob. > \|z\|** |
| --- | --- | --- | --- | --- |
| Type of misallocation |  |  |  | 0.000 |
| - Not sent DVD in DVD week | Ref. |  |  |  |
| - Sent DVD in no DVD week | 0.42 | (0.33-0.52) | -7.95 |  |
| Sex |  |  |  | 0.108 |
| - Female | Ref. |  |  |  |
| - Male | 1.17 | (0.97-1.43) | 1.61 |  |
| Ethnicity |  |  |  | 0.974 |
| - Pacific | Ref. |  |  |  |
| - Māori | 1.00 | (0.82-1.23) | 0.06 |  |
| Age at follow-up date (years) | 1.01 | (0.99-1.02) | 1.04 | 0.298 |
| Deprivation quintile |  |  |  | 0.051 |
| - Q1 (least) | Ref. |  |  |  |
| - Q2 | 0.74 | (0.50-1.10) | -1.50 |  |
| - Q3 | 0.64 | (0.44-0.92) | -2.42 |  |
| - Q4 | 0.58 | (0.41-0.82) | -3.12 |  |
| - Q5 (most) | 0.61 | (0.43-0.88) | -2.68 |  |
| - Missing | 0.67 | (0.44-1.02) | -1.87 |  |

Note: Variables tested for significance differences between groups using the Wald test.

# Effect of DVD on Participation with Non-Spoiled Kit

**Table S2** shows the differences in unspoiled kit return rate which are smaller than those for kit return rates overall, reflecting the association between being sent a DVD and returning a non-spoiled kit. Being sent a DVD was associated with a significantly lower rate of return of an unspoiled kit (on first attempt) for all but Pacific females.

**Table S2.** Return rates of unspoiled kits in DVD and No DVD groups by sex and ethnicity.

|  | **DVD Group** | **Unspoiled kit returned** | **Kit not returned** | **Total** | **Unspoiled kit return rate** | **Difference (95% C.I.)** |
| --- | --- | --- | --- | --- | --- | --- |
| **Māori** | **Sent DVD** | 120 | 892 | 1012 | 11.9% | 5.5%  (2.6%-8.3%) |
|  | **Not sent DVD** | 226 | 1078 | 1304 | 17.3% |  |
| **Pacific** | **Sent DVD** | 105 | 1224 | 1329 | 7.9% | 2.7%  (0.1%-4.8%) |
|  | **Not sent DVD** | 168 | 1411 | 1579 | 10.6% |  |
| **Māori male** | **Sent DVD** | 47 | 343 | 390 | 12.1% | 6.9%  (2.1%-11.7%) |
|  | **Not sent DVD** | 104 | 445 | 549 | 18.9% |  |
| **Māori female** | **Sent DVD** | 73 | 549 | 622 | 11.7% | 4.4%  (0.1%-8.1%) |
|  | **Not sent DVD** | 122 | 633 | 755 | 16.2% |  |
| **Pacific male** | **Sent DVD** | 40 | 549 | 589 | 6.8% | 5.1%  (2.0%-8.2%) |
|  | **Not sent DVD** | 85 | 630 | 715 | 11.9% |  |
| **Pacific female** | **Sent DVD** | 65 | 675 | 740 | 8.8% | 0.1%  (-2.0%-3.7%) |
|  | **Not sent DVD** | 83 | 781 | 864 | 9.6% |  |

Note: Excludes subjects with missing gender.

# Outcome Results Based on Randomisation Intention

When analysed according to the randomisation intention (DVD/NO DVD week), rather than by protocol (Sent or Not Sent a DVD), there is no significant effect observed (**Table S3**). The misallocation of protocol in some of the subjects hides the effect of the DVD.

**Table S3.** Kit return rates in DVD and No DVD weeks (analysis by randomisation intention)

|  | **DVD Group** | **Kit returned** | **Kit not returned** | **Total** | **Kit return rate** | **Difference (95% C.I.)** |
| --- | --- | --- | --- | --- | --- | --- |
| **Māori** | **DVD week** | 229 | 887 | 1116 | 20.5% | 0.1%  (-3.2%-3.4%) |
|  | **No DVD week** | 247 | 953 | 1200 | 20.6% |  |
| **Pacific** | **DVD week** | 203 | 1237 | 1440 | 14.1% | 1.0%  (-1.6%-3.5%) |
|  | **No DVD week** | 221 | 1247 | 1468 | 15.1% |  |
| **Māori male** | **DVD week** | 99 | 350 | 449 | 22.0% | 1.6%  (-3.7%-7.0%) |
|  | **No DVD week** | 116 | 374 | 490 | 23.7% |  |
| **Māori female** | **DVD week** | 130 | 537 | 667 | 19.5% | -1.0%  (-5.2%-3.1%) |
|  | **No DVD week** | 131 | 579 | 710 | 18.5% |  |
| **Pacific male** | **DVD week** | 92 | 555 | 647 | 14.2% | 2.1%  (-1.8%-6.0%) |
|  | **No DVD week** | 107 | 550 | 657 | 16.3% |  |
| **Pacific female** | **DVD week** | 111 | 682 | 793 | 14.0% | 0.1%  (-3.3%-3.5%) |
|  | **No DVD week** | 114 | 697 | 811 | 14.1% |  |

Note: Excludes subjects with missing gender.

**Table S4.** Analysis of kit return rates in correctly allocated subjects only.

In **Table S4** incorrectly allocated subjects have been removed from the analysis. The negative effect of the DVD on participation is significant for both Māori and Pacific and all gender subgroups. These results are consistent with those presented in **Table 2** which analysed the effect of being sent a DVD in all subjects, although the magnitude of the difference is somewhat smaller in each group, showing that being sent a DVD had at least as strong an effect in those who were incorrectly allocated the intervention as in those who received the intervention according to the intended randomisation.

|  | **DVD Group** | **Kit returned** | **Kit not returned** | **Total** | **Kit return rate** | **Difference (95% C.I.)** |
| --- | --- | --- | --- | --- | --- | --- |
| **Māori** | **DVD in DVD week** | 131 | 829 | 960 | 13.6% | 7.3%  (4.1%-10.5%) |
|  | **No DVD in no DVD week** | 240 | 908 | 1148 | 20.6% |  |
| **Pacific** | **DVD in DVD week** | 131 | 1130 | 1261 | 10.4% | 5.2%  (2.6%-7.7%) |
|  | **No DVD in no DVD week** | 218 | 1182 | 1400 | 15.6% |  |
| **Māori male** | **DVD in DVD week** | 48 | 325 | 373 | 12.9% | 10.8%  (5.7%-15.9%) |
|  | **No DVD in no DVD week** | 112 | 361 | 473 | 23.7% |  |
| **Māori female** | **DVD in DVD week** | 83 | 504 | 587 | 14.1% | 4.8%  (0.7%-8.9%) |
|  | **No DVD in no DVD week** | 128 | 547 | 675 | 18.9% |  |
| **Pacific male** | **DVD in DVD week** | 53 | 505 | 558 | 9.5% | 7.3%  (3.5%-11.1%) |
|  | **No DVD in no DVD week** | 105 | 521 | 626 | 16.8% |  |
| **Pacific female** | **DVD in DVD week3** | 78 | 625 | 703 | 11.1% | 3.5%  (0.1%-6.9%) |
|  | **No DVD in no DVD week** | 113 | 661 | 774 | 14.6% |  |

Note: Excludes subjects with missing gender.
